# Supplementary material for: Endoplasmic Reticulum Stress-Related Ten-Biomarker Risk Classifier for Survival Evaluation in Epithelial Ovarian Cancer and TRPM2: A Potential Therapeutic Target of Ovarian Cancer
Source: Int J Mol Sci. 2023 Sep 12;24(18):14010. doi: 10.3390/ijms241814010 (PMC10530916; doi:10.3390/ijms241814010)
Supplement: Supplementary file 1 [file ijms-24-14010-s001.zip › ijms-2561881-supplementary.pdf]

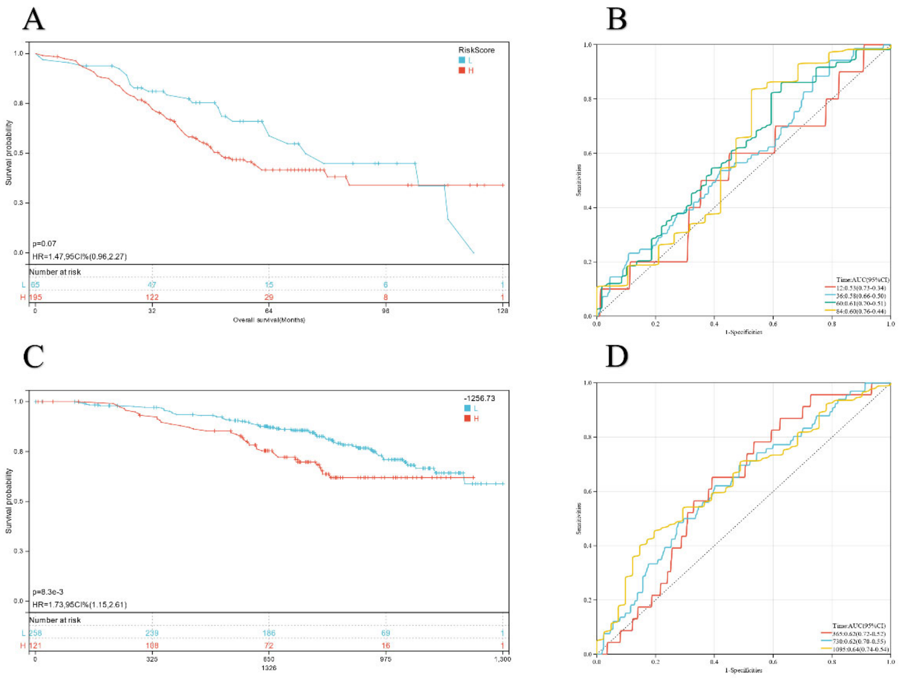

Supplementary Figure S1. (A) The outcome of survival analysis of the risk scores in the GSE32026 dataset; (B) The outcome of ROC analysis of the risk scores in the GSE32026 dataset; (C) The outcome of survival analysis of the risk scores in the GSE140082 dataset; (D) The outcome of ROC analysis of the risk scores in the GSE140082 dataset.
